# A profile of deaths among trauma patients in a university hospital: The Philippine experience

**DOI:** 10.5249/jivr.v3i2.39

**Published:** 2011-07

**Authors:** Rafael J. Consunji, John Paul Emerson Serrato Marinas, Jason Rafael Aspuria Maddumba, Daniel A. Dela Paz Jr.

**Affiliations:** ^*a*^Division of Trauma, Department of Surgery, Philippine General Hospital, College of Medicine, University of the Philippines, Manila, Philippines.; ^*b*^Study Group on Injury Prevention and Control, National Institutes of Health, University of the Philippines, Manila, Philippines.

## Abstract

**Background:**

The Philippine General Hospital (PGH) is the pioneer in trauma care in the country, being the first to create a dedicated Trauma Service in 1989. The service has not conducted a review of its admissions and mortalities since 1992. The purpose of this study is to describe the mortality patterns of this service.

**Methods:**

A descriptive and retrospective 3-year review, covering January 2004 June 2007, was conducted using an electronic patient database. Review of patient records included: population demographics, mechanism of injury, length of stay prior to death, and the cause of death.

**Results:**

Of the 4947 patients admitted to the Division of Trauma during the study period, there were 231 (4.7%) deaths. The most common mechanisms of injuries were stab wounds (32.9 %), vehicular crashes (28.6 %), and gunshot wounds (25.5 %). Multiple organ failure/Sepsis (37.7 %) was the most frequent causes of death, followed by Exsanguinations (27.7 %), Central Nervous System failure (18.6 %) and other causes (10.8%). Forty four (66.7 %) of the 66 patients who died within the first 24 hours died from Exsanguinations, while 66 (61.1 %) of the 8 patients who died after 72 hours died from Multiple organ failure/Sepsis.

**Conclusions:**

Intentional causes of injury (i.e. penetrating interpersonal violence) caused the majority of trauma deaths in this series from the Philippine General Hospital. This highlights the need for prioritizing a public health approach to violence prevention in the Philippines. Further research must be conducted to identify risk factors for interpersonal violence. Early identification of lethal injuries that may cause exsanguinations and definitive control of hemorrhage should be the primary focus to prevent acute deaths, within 24 hours of admission. Further adjuncts to the definitive treatment of hemorrhage, the critical care of TBI and MOF/Sepsis are needed to reduce deaths occurring more than 72 hours after admission.

## Introduction

Accidents (external causes of death), injuries from hereon, are one of the leading causes of mortality in the Philippines, presently they are the 4th leading cause of death for all ages. Philippine Health Statistics reports, for the years 1975 to 2002, indicate that injury mortality rates  have more than doubled from 19.1 in 1975 to 41.9 in 2003 (per 100,000 population). The top five leading causes of death due to injury in the Philippines, for all ages, are assault, transport accidents, accidental drowning and submersion, intentional self-harm and accidental falls.^1^

In response to these trends, the Philippine Department of Health included injury prevention in the National Objectives for Health (2005-2010).  Injury specific objectives include: ‘mortality secondary to accidents should be reduced to 40 deaths per 100,000 populations and those secondary to transport accidents should be reduced to 6 deaths per 100,000 population’ by 2010. Presently, national data stands as 42.4 and 7.4 respectively, based on the most recent available Philippine Health statistics.^2^

Accurate mortality statistics are important to inform health policy by serving as a basis for implementing appropriate prevention strategies, improving emergency preparedness, and instituting financing policies and appropriate health packages. These data must come from multiple sources, census and mortality reports, hospital databases and community-based reports in order to increase its validity and reduce under reporting. Philippine health statistics for mortality and disability from injury are still wanting.^2^One of the current challenges for the Department of Health is to consolidate and improve the quality of its data on the burden of disease from injury in order to increase the likelihood of meeting its National Objectives of Health. None of theses objectives have ‘trickled’ down to the instiutional level, in terms of trauma center acreditation, research or training.

The Division of Trauma, Department of Surgery, Philippine General Hospital is the pioneer in trauma care in the country, being the first dedicated Trauma Service tending specifically to those with severe and multiple injuries.  The PGH trauma unit is located within the City of Manila (the most urban and dense city within the National Capital Region also known as Metropolitan Manila with a population of 11-12 million people and a density of 19,000 persons/km2).^3^It admits about 1600 patients every year, and has no assigned trauma catchment area, geographic jurisdiction or triage area. Known as the National University Hospital, PGH receives most of its patients from the lower socio-economic population who take advantage of the minimal fees it charges for services rendered. Organized pre-hospital emergency medical service (EMS) systems are almost non-existent in the Philippines, with most trauma cases being ‘self-conducted’ to the nearest trauma center. At the time of this study, there was no ‘trauma leveling’ or classification system for the level of care that hospitals in the Philippines can provide.

Institutionally, the Division of Trauma, Department of Surgery, Philippine General Hospital, University of the Philippines, Manila (PGH) has been instrumental in implementing the latest advances in trauma care and being a leader in continuing improvement and education of trauma care providers nationwide since its inception in 1989. However, no recent data from PGH is available to describe the characteristics of the trauma population, specifically that of the fatalities.

The purpose of this study is to describe the mortality patterns of the Trauma Service at the Philippine National University Hospital. Specifically, the authors aim to identify pattern of deaths according to: a) mechanism of injury; b) age; c) cause of death; and d) time of death. Understanding the predictive factors that identify patients who are at an increased risk of trauma mortality can improve the delivery of patient care, direct research efforts, target staff education programs, and guide injury prevention activities.

## Methods

A retrospective review, of medical records, was performed on all patients admitted by the Division of Trauma, Department of Surgery, Philippine General Hospital from January 2004 – May 2007. Specific data was collected on all patients who died, as inpatients, while admitted to this service.

**Figure 1: Trauma deaths, by Mechanism, PGH(04-07), all ages:**
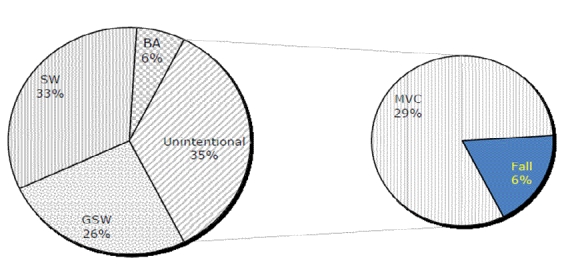
PGH: The Philippine General Hospital, GSW: gunshot wound, SW: stab wound,  MVC: motor vehicle crash, BA: blunt assault

Data was collected from the Integrated Surgical Information System (ISIS) of the Department of Surgery, an electronic and online database that has been in use since January 1, 2004. ISIS is an online database of surgery patients that provides convenient organization and retrieval of medical files, with medical clerks and interns in charge of data input. The system is limited to patients admitted to the hospital; emergency room consults and deaths are not entered into the ISIS. Data collected for each traumatic death included in the study consisted of the cause of death; time of death from time of admission; mechanism of injury; demographics and age at the time of admission.Inclusion criteria specified that trauma deaths must have occured after admission to the hospital. Excluded from analysis were deaths caused by drowning, poisonings, or burns as these traumatic injuries were handled by other non-trauma services. Likewise, patients with isolated burns, neurologic, orthopedic, ophthalmologic or otorhinolar-yngologic injuries excluded in this profile of traumatic deaths.(Figure 1)

Mechanisms of injury, as indicated in the patients’ medical records, were grouped as stab wound (SW), gunshot wound (GSW), motor vehicle crash (MVC), falls and blunt assault (BA).  This set of data was cross-referenced with age groups of the patients. Age groups were clustered into 4 groups: ≤20, 20 to 40, 40 to 60 and ≥60 years old, as per Demetriades et al.^4^

The antecedent cause of death, was determined by the consensus of Trauma Attending Staff during weekly trauma team conferences, it was classified into five categories, modified from Sauaia et al. 1995,^5^CNS, Exsanguination, MOF/Sepsis, Others and Undetermined.

Data on the cause of death were classified according to the time of death from admission/consultation at PGH till pronouncement of death. Time to death was classified as acute (within 24 hours of admission), early (within 24 to 72 hours), and late (>72 hours). No pre-hospital deaths, deaths on arrival or ER deaths were included in this study.

## Results

Of the 4947 patients admitted to the Division of Trauma during the study period, there were 231 (4.7%) deaths, 205 (88.7 %) of these were males. The mean age of fatalities was 33.5 yrs.

For the underlying mechanism of death for all ages, 135 (58.4 %) were victims of penetrating injuries (GSW's in 59, SW's in 76), and 96 (41.6%) had blunt trauma (vehicular injury in 66, falls in 15, mauling in 5, and other blunt injuries in 10). Intentional causes of injury (stab wounds, gunshot wounds and blunt assault) led to 151 (65%) deaths, while unintentional causes (vehicular crashes and falls) caused 80 (35%) deaths.

Over half of all trauma deaths occurred in young adults, ages 20-40 (51.5% of deaths). For children-adolescents and the older adult groups (1-20 and >60), unintentional injuries (primarily MVC’s) were the most common mechanism of lethal trauma. Intentional injuries caused the most trauma deaths for the ages 20-60 with stab wounds as the primary cause (Table 1).

**Table:** Table 1: **Trauma deaths, by mechanism and age group, PGH (2004-2007)**

Age group					All ages (231, 100%)	0--20 yrs.(41 - 17.7%)	20-40 yrs. (119 - 51.5%)	40-60 yrs. (60 - 26.9%)	>60 yrs. (11 - 4.8%)
					All ages	0-20 yrs	20-40 yrs	40-60 yrs	> 60 yrs
Mechanism	n	%	n	%	n	%	n	%	n	%
GSW	59	25.5%	9	22.0%	35	29.4%	15	25.0%	0	0.0%
SW	76	32.9%	9	22.0%	49	41.2%	19	31.7%	1	9.1%
MVC	66	28.6%	15	36.6%	34	28.6%	19	31.7%	8	72.7%
Fall	15	6.5%	2	4.9%	6	5.0%	5	8.3%	2	18.2%
BA	15	6.5%	6	14.6%	7	5.9%	2	3.3%	0	0.0%
Total	231	100.0%	41	17.7%	119	51.5%	60	26.0%	11	4.8%

PGH: The Philippine General Hospital, GSW: gunshot wound,  SW: stab wound, MVC: motor vehicle crash,  BA: blunt assault

Overall, MOF/Sepsis was the most common antecedent cause of death, caused primarily by sepsis (42.5 %) and ARDS (29.9 %) with exsanguination as a close second. A CNS cause of death was third (43 or 18.6 %), with the direct sequelae of traumatic brain injury (63.4 %) and hypoxic encephalopathy (36.6 %). The causes of death of 12 (5.2 %) patients remained undetermined, all due to incomplete medical records. Almost half of all trauma deaths occurred >72 hours after admission, mostly from MOF/Sepsis. Two-thirds of the acute deaths, within the first 24 hours, occurred as a result of exsanguination, with MOF/Sepsis in the lead among the closely grouped causes of death within 24-72 hrs of admission (Table 2).

**Table:** Table 2: **Antecedent cause of death in relation to interval to death **

	<24 hrs	24-72 hrs	>72 hrs	Total
CNS	19.7	26.3	13.9	18.6
Exsanguination	66.7	21.1	7.4	27.7
MOF/Sepsis	1.5	35.1	61.1	37.7
Others	9.1	10.5	12	10.8
Undetermined	3	7.0	5.6	5.2
Total	28.5	24.7	46.8	100

CNS: central nervous system,  MOF: multiple organ failure

## Discussion

This is the first study to describe the pattern of in-hospital trauma fatalities in PGH, the National University Hospital of the Philippines. This findings highlight the need to prioritize violence prevention, set the benchmark for the quality assessment of trauma care and identify areas for the tertiary prevention of trauma deaths and disability.

While the provision of organized trauma care is in its infancy in many parts of the Philippines, the PGH Trauma Service has been providing care for twenty years since its inception in 1989. It has demonstrated improved outcomes in its early years, resulting in improved survival and a reduced complication rate among trauma patients.^6^Since then the service has treated more than 21,000 trauma admissions, this study describes the fatalities amongst 4,947 admissions from January 2004 to May 2007.

Overall, MOF/Sepsis was the leading cause of trauma fatalities in this series. Exsanguinations from intentional penetrating injuries was the most common cause of acute trauma death within 24 hours of admission to the PGH Division of Trauma, with MOF/Sepsis as the leading cause of late trauma deaths (>72 hours after admission). Young male victims of stab or gunshot wounds made up the majority of trauma mortalities.

This series was culled from a database of almost 5,000 trauma admissions at the major trauma referral hospital in the Philippines, making this a more than adequate representation of local, major multi-system trauma. While the data was gathered retrospectively, the relative completeness and accessibility of the electronic database demonstrated the utility of such for the conduct of initial, seminal research specifically to identify gaps within the database and guide future areas for research. Some of these findings are unique, not lending them to easy extrapolation to dissimilar settings because of the predominance of penetrating torso trauma (~65%) and the exclusion of single system or isolated trauma (i.e. TBI due to cranial trauma).

The evidence to classify the antecedent cause of death was taken from case records and a modified Deplhi technique, using a modified classification based on Sauia.^5^A ‘real-time’ or immediate classification of the antecedent cause of death, using a standard template, would be an improvement in this adjudication process. This will be the focus of subsequent research at our center.

The data on the victims and mechanisms of trauma deaths does not differ in pattern dramatically from those of other local authors, who also report a young, male predominance with majority as victims of intentional penetrating injury.^7^7 However, the pattern of trauma deaths is quite distinct from those reported by others;^4,5,8,15^this is so for a number of reasons:

**1. The database excludes victims of isolated neurologic trauma.**

The PGH Trauma Service is activated for all instances of multi-system trauma, affecting > 1 region and needing care from > 1 surgical specialty service, and for major torso trauma. This explains the variance this study has with others who report that CNS injuries make up the majority of acute trauma deaths.^5,8^

**2. The inadequacy of organized pre-hospital services for trauma.**

Pre-hospital care in the Philippines is largely non-existent for trauma patients. In a review of pediatric trauma ER consults at PGH, it was noted that only 4% of cases were seen by a physician prior to arrival, 7% were referred from another hospital and 79% were self-transported to the hospital. Only 25% of patients arrived in less than 60 minutes from injury, 37% from 1-4 hours and 9% more than 48 hours from time of injury.^9^This, along with the inability to extract the true time of injury from the medical records, explains the definition of time interval to death used.

The relatively low over-all mortality rate (4.7%) is reflective of the ‘self-triage’ or ‘self-selection’ of severe trauma in the Philippines; those with very severe injuries or injured at distances too far to arrive prior to demise will succumb to their injuries before arriving at a hospital.

**3. The high prevalence of penetrating and intentional injury in this database.**

The fact that 60% and 65% of all trauma deaths can be attributed to penetrating and intentional injuries renders this dataset unique when compared to trauma centers in high-income, even urban, trauma centers.^4^

Sauaia et al. and Meislin et al., reported gunshot wounds to be the most common cause of deaths, followed by vehicular crashes.^5,8^Our data showed a similar mortality pattern with penetrating trauma as the leading trauma killer, followed by blunt injuries sustained in MVC's. The major difference is in that penetrating injuries in the Philippines are most commonly inflicted with knives, rather than guns.^10-12^Trunkey and Blaisdell combined data from the 1977 Baker study with those of a 1974 epidemiologic study also done by their group to derive the often-cited “trimodal distribution of trauma deaths'' that has served as the impetus for the development of most trauma systems.^8^The first peak included immediate deaths that are primarily the result of CNS and exsanguinations from major vascular trauma. The second peak included the early hospital deaths that occurred within a few hours after injury, principally caused by CNS injuries and exsanguinations. The third peak constituted the late deaths, majority of which were caused by MOF/sepsis.

This study observed a bimodal pattern, but with limitations, since the first peak, which constitutes pre-hospital deaths, was excluded. The absence of an organized EMS system in Metro Manila explains the lack of data on pre-hospital deaths from trauma in the Philippines. There is a 'natural selection' of trauma victims only those with less severe or slower exsanguination arrive alive at major trauma centers. Early hospital deaths were primarily caused by exsanguination, while late deaths were mostly attributable to MOF. The number of deaths caused by CNS injuries remained relatively constant throughout the duration of stay.

From a primary prevention standpoint, the data emphasizes the importance of intentional injury and violence prevention in the Philippine highlighting the need for further research to ascertain the risk factors, such as alcohol, drugs or other predisposing elements to interpersonal violence, the leading mechanism of trauma deaths.

In the Philippines, it is not uncommon for a trauma victim to be diverted through^1-2^different hospitals before locating a medical center willing to accept him, often incurring more blood loss and decrements in his GCS. This loss of the proverbial ‘golden hour’ of trauma care in the Philippines has not yet been adequately studied and this should be the focus of further research. These places the onus of quality assurance for trauma care squarely on the shoulders of trauma care providers in trauma centers because only those with highly ‘survivable’ injuries will arrive to seek care. 

From a trauma care standpoint, the early identification of lethal injuries that may cause exsanguinations and subsequent definitive control of hemorrhage should be the focus to prevent acute and early deaths.^13^Further adjuncts to the definitive treatment of hemorrhage (i.e. improved haemostatic techniques, through surgery and interventional radiology, massive blood transfusion protocols, increased availability of blood and its components and bedside coagulation testing capabilities in the operating rooms),^14^identification of an earlier arrival at trauma designated centers, and the critical care of TBI (Neuro ICU beds), MOF and sepsis (ICU beds and support services) are needed^15^if reductions in trauma deaths are the goal.

Steps must also be taken to improve injury surveillance and the quality of data collected. Detailed, complete and relevant data will guide prevention efforts aimed at risk factors in the individual and the environment^15^and provide feedback to trauma care providers. Further monitoring of these trends will influence training, improve the focus of the trauma service and direct the provision of more effective care to these severely injured patients.
